# Complete Genome Sequence of *Acetobacter pomorum* Oregon-R-modENCODE Strain BDGP5, an Acetic Acid Bacterium Found in the *Drosophila melanogaster* Gut

**DOI:** 10.1128/genomeA.01333-17

**Published:** 2017-11-30

**Authors:** Kenneth H. Wan, Charles Yu, Soo Park, Ann S. Hammonds, Benjamin W. Booth, Susan E. Celniker

**Affiliations:** Biological Systems and Engineering Division, Lawrence Berkeley National Laboratory, Berkeley, California, USA

## Abstract

*Acetobacter pomorum* Oregon-R-modENCODE strain BDGP5 was isolated from *Drosophila melanogaster* for functional host-microbe interaction studies. The complete genome is composed of a single chromosomal circle of 2,848,089 bp, with a G+C content of 53% and three plasmids of 131,455 bp, 19,216 bp, and 9,160 bp.

## GENOME ANNOUNCEMENT

In *Drosophila*, *Acetobacter* spp. are among the major commensals of the gut microbiota and contribute to larval growth (reviewed in reference 1). Furthermore, monocolonization of *Drosophila* with *Acetobacter* species significantly reduced host development (2). The first draft sequence of *Acetobacter pomorum* from *Drosophila*, published in 2014, consisted of 137 contigs (3). We report here the sequence of the complete genome, consisting of a single chromosome and three plasmids.

*A. pomorum* Oregon-R-modENCODE strain BDGP5 was isolated from homogenized dissected guts. Bacteria were streaked onto Nutrient broth agar (BD catalog number 213000) plates, single colonies were amplified in culture, and an aliquot was used for 16S V1 and V4 PCR (4) and sequence identification (reviewed in reference 5). DNA for sequencing was isolated (6), and whole-genome DNA sequencing was performed by the National Center for Genome Resources (NCGR) (Santa Fe, NM), using Pacific Biosciences (PacBio) long reads on the RSII instrument (7). A single-molecule real-time (SMRT) cell library was constructed with 5 to 10 µg of DNA using the PacBio 20-kbp protocol. The library was sequenced using P6 polymerase and C4 chemistry with 6-h movie times. Sequencing yielded a total of 52,913 reads with filtered mean read length of 12,181 bp, totaling 644,585,547 bp (>150-fold coverage). A *de novo* assembly was constructed using the Hierarchical Genome Assembly Process (HGAP2) protocol from SMRT Analysis version 2.0 (8–10). The final contigs were manually trimmed and reviewed to produce a single circular chromosome and three plasmids. Annotations of protein-coding open reading frames and noncoding RNAs (ncRNAs) were predicted using the Rapid Annotations using Subsystems Technology (RAST) tool (11) and the GenBank annotation pipeline (12).

The chromosomal genome annotation predicted 2,858 genes in total, with 2,782 protein-coding genes, 77 RNA genes, including 5 rRNA operons, 57 tRNA genes, 1 transfer-messenger RNA (tmRNA) gene, 3 noncoding RNA (ncRNA) genes, and 120 pseudogenes. Of the 2,782 protein-coding genes, 46 are contained within two partial cryptic prophages (20 kb, 24 genes; 17.7 kb, 22 genes). The first is characterized by a number of genes encoding Mu-like FluMu proteins (13). The candidate prophages are only 1.3% of the genome. Additionally, the genome contains three plasmids, pApBDGP5A (151,013 bp), pApBDGP5B (9,160 bp), and pApBDGP5C (19,216 bp), with predicted G+C contents of 52%, 54%, and 54%, respectively. The plasmids contain 203 candidate protein-coding genes. Among them are genes essential for conjugation (*traG* [pApBDGP5A]), plasmid replication (*repA* [pApBDGP5A and pApBDGP5C]), and toxin-antitoxin (TA) modules associated with stable plasmid inheritance at cell division, including *relE/stbE-relB/stbD* (pApBDGP5A) *doc-phd*, *yoeB-yefM,* and *vapC-B* (pApBDGP5C) (reviewed in reference 14).

Intriguingly, the bacterial chromosome also contains toxin-antitoxin modules for *doc-phd*, *mazF-E*, *vapC-B*, and *hicA-B*, thought in the chromosomal case to be important for bacterial stress response (reviewed in reference 15).

Our sequence has significant similarity to *Acetobacter pasteurianus*, being 98.8% identical by average nucleotide identity (ANI) (16). Despite the sequence similarity, phenotypically, our strain belongs to the *A. pomorum* species based on its ability to grow in *n*-propanol and glycerol (17).

### Accession number(s).

The complete chromosome and three plasmid sequences of *A. pomorum* Oregon-R-modENCODE strain BDGP5 are deposited in GenBank under the accession numbers CP023657 (chromosome), CP023658 (plasmid pApBDGP5A), CP023659 (plasmid pApBDGP5B), and CP023660 (plasmid pApBDGP5C).
